# Association among cytotoxic T-lymphocyte antigen 4 gene, rs231775 polymorphism, and recurrent pregnancy loss risk

**DOI:** 10.1042/BSR20181760

**Published:** 2019-02-01

**Authors:** Yonghui Song, Ying Chen, Qian Xu

**Affiliations:** Department of Obstetrics, Linyi People’s Hospital, Linyi 276003, Shandong, China

**Keywords:** Cytotoxic T-lymphocyte antigen 4, meta-analysis, polymorphism, recurrent pregnancy loss

## Abstract

Cytotoxic T-lymphocyte antigen 4 (*CTLA-4*) is expressed constitutively on regulatory T cells. So far, several studies have focused on association between *CTLA-4* gene polymorphisms and recurrent pregnancy loss (RPL). However, above association between the *CTLA-4* gene polymorphism and RPL susceptibility is uncertain. Therefore, we performed a timely meta-analysis of all current publications to clarify this relationship. We located articles from the PubMed and Chinese language (WanFang) databases that were published up until July 25, 2018. Finally, we obtained six case–control studies, containing 2405 total cases and 2607 total controls, based on search criteria for abortion susceptibility related to the *CTLA-4* +49 G/A polymorphism. The odds ratios (OR) and 95% confidence intervals (CIs) revealed association strengths. There was significantly decreased association between this polymorphism and whole population risk (e.g. AA vs. GG: OR = 0.56, 95% CI = 0.38–0.81, *P*=0.002). Additionally, in ethnicity subgroups, similar association was found both in China (e.g. AA vs. GG: OR = 0.49, 95% CI = 0.39–0.63, *P*=0.002) and non-China (e.g. AG vs. GG: OR = 0.46, 95% CI = 0.34–0.63, *P*<0.001). Current analysis suggested *CTLA-4* +49 G/A polymorphism may weakly decrease RPL risk for women of childbearing age.

## Introduction

A pregnancy loss (PL) is defined as the spontaneous demise of a pregnancy before the fetus reaches viability. It includes all PLs [unexplained recurrent spontaneous abortion (RSA), RSA, recurrent miscarriage (RM), idiopathic RM] from the time of conception until 24 weeks of gestation [1,2]. Approximately 15% of pregnant women experience sporadic loss, 2% experience two consecutive PL and 0.4–1% have three consecutive PL [3]. Recurrent PL (RPL), also named as recurrent spontaneous abortion (RSA), is defined as the loss of two or more pregnancies [1,2,4]. In addition, RM is classically defined as the loss of three or more consecutive pregnancies before the 20th weeks of gestation with or without previous live births [5]. In the same time, the definition of RPL and RM exists some discrepancy in opinions, broadly speaking, both are classified as PL or abortion.

A series of pathogenic mechanisms associated with PL has been described, including uterine abnormalities, endocrine and metabolic problems, genetic anomalies, acquired and inherited thrombophilia and immunological factors [6]. More and more studies have focused on the genetic factors, especially the single nucleotide polymorphism (SNP) [7].

The cytotoxic T-lymphocyte antigen 4 (*CTLA-4*, Gene ID: 1493, MIM number: 123890 also known as GSE, ALPS5, CD152) gene maps to band q33 of human chromosome 2, spans about 6.2 kilobases, and contains four exons and three introns [8]. It is well known that *CTLA-4* expressed on human placental regulatory T (T_reg_) cells in decidual and peripheral dendritic cells may induce the expression of an immune-suppressive enzyme indoleamine 2,3-dioxygenase (ID), particularly during early phases of pregnancy [9]. Furthermore, high expression of ID promotes maternal-fetal tolerance [9]. In addition, the expression of T_reg_ cells and *CTLA-4* in peripheral and decidual lymphocytes was down-regulated in human miscarriages in several *in vivo* studies [10]. We predicted *CTLA-4* and its related T_reg_ cells are protective factors for RPL. SNPs are known as the most common type of DNA variation in individuals [11], which may affect DNA promoter activity and influence the translation, and finally may be associated with the susceptibility about human diseases [11]. Therefore, we hypothesized that the reduced number and/or functional deficiency of T_reg_ cells due to the genetic variations in *CTLA-4* gene may increase the risk of RPL.

So far, many studies have investigated the association between *CTLA-4* rs231775 G/A polymorphism (wild-type allele: A; polymorphic allele: G, 49A>G, Thr17Ala) and RPL risk. However, the results were not conclusive or consistent. Thus, we conducted this timely meta-analysis of six case–control studies to derive a more powerful estimation of the association between *CTLA-4* rs231775 G/A polymorphism and RPL susceptibility [12–17].

## Materials and methods

### Identification and eligibility of relevant studies

Searches were conducted in PubMed and Chinese language (WanFang) databases using the key words ‘cytotoxic T-lymphocyte antigen 4 or *CTLA-4’*, ‘spontaneous abortion or miscarriage or pregnancy loss’ and ‘polymorphism’ or ‘variant’. The last search was updated on July 25, 2018. In total, 18 articles were retrieved using the abovementioned terms, and six articles contained the inclusion criteria.

### Inclusion criteria and exclusion criteria

Including studies had to meet following criteria: (1) address the correlation between RPL risk and the *CTLA-4* rs231775 G/A SNP; (2) be a case–control study; and (3) have sufficient numbers of genotypes (AA, AG, and GG) for both the cases and controls. The following exclusion criteria were used: (1) lack of a control population; (2) lack of available genotype frequency data; and (3) duplicated studies.

### Data extraction

The following items were collected: the last name of first author, the year of publication, the country of origin, the ethnicity of subjects, source of control (SOC), the total and number of each genotype frequency in the case–control groups, the Hardy–Weinberg equilibrium (HWE) of the controls, abortion type, control-type and the genotyping method. Ethnicity was categorized as Asian, China, and non-China.

### Quality score assessment

The quality score assessment (Newcastle–Ottawa Scale, NOS) [18] was selected to assess the quality of each study. This measure assesses aspects of the methodologies used in observational studies, which are related to the study quality, including selection of cases, comparability of populations, and ascertainment of exposure to risks. The NOS rating ranges from zero stars (worst) to nine stars (best). Studies with a score of seven stars or greater was considered as a high quality.

### Statistical analysis

Odds ratios (OR) with 95% confidence intervals (CI) were used to measure the strength of the association between the *CTLA-4* rs231775 G/A SNP and RPL risk. The statistical significance of the summary OR was determined with the *Z*-test. A heterogeneity assumption was evaluated among studies using a chi-square-based *Q*-test. A *P-*value of more than 0.10 for the *Q*-test indicated a lack of heterogeneity among the studies [19]. If significant heterogeneity was detected, the random-effects model (DerSimonian–Laird method) was used. Otherwise, the fixed-effects model (Mantel–Haenszel method) was chosen [20,21].

We investigated the relationship between genetic variants of the *CTLA-4* rs231775 G/A site and RPL risk by the allelic contrast (A-allele vs. G-allele), homo*z*ygote comparison (AA vs. GG), dominant genetic model (AA+AG vs. GG), heterozygote comparison (AG vs. GG), and recessive genetic model (AA vs. AG+GG). A sensitivity analysis was performed by omitting studies, one after another, to assess the stability of results. The departure of the *CTLA-4* rs231775 G/A SNP from expected frequencies under HWE was assessed in controls using the Pearson chi-square test (*P* < 0.05 was considered significant). Funnel plot asymmetry was assessed using Begg’s test, and publication bias was assessed using Egger’s test [22], both of *P*-value less than is considered as significant. All statistical tests were performed using STATA Software (version 11.0; StataCorp LP, College Station, TX).

### Network of gene interaction of *CTLA-4* gene

The network of gene–gene interaction for *CTLA-4* gene was utilized through String online server (http://string-db.org/) [23].

## Results

### Study characteristics

**Figure 1:**
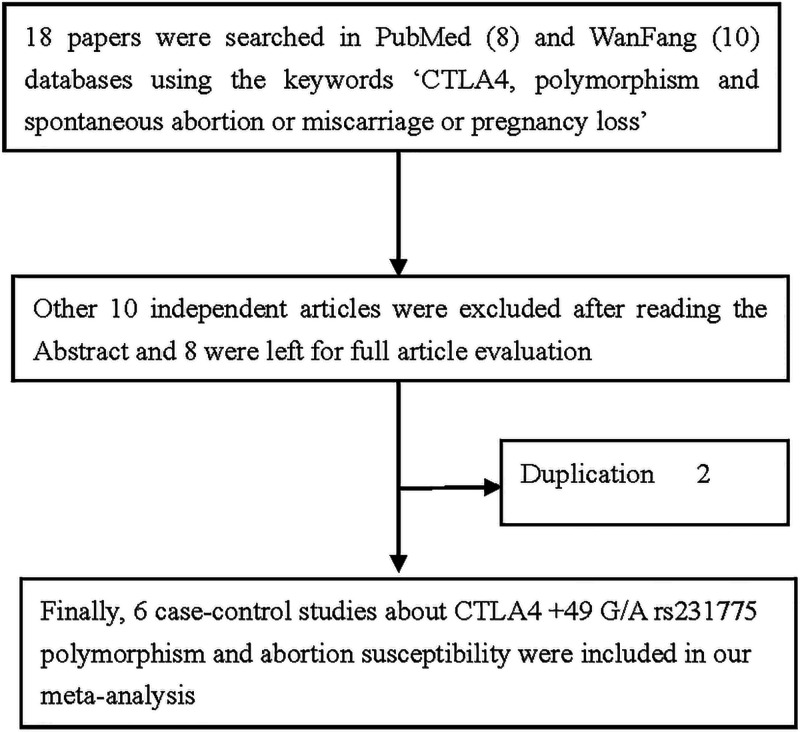
Flowchart illustrating the search strategy used to identify association studies for *CTLA-4* gene rs231775 polymorphism and RPL risk

**Figure 2:**
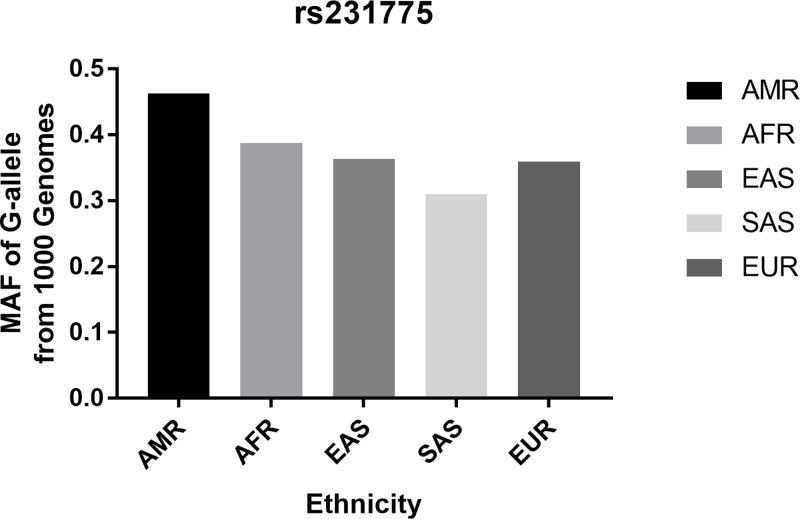
A-allele frequencies for the *CTLA-4* gene rs231775 polymorphism among cases–controls stratified by ethnicity Vertical line, T-allele frequency; horizontal line, ethnicity type. Abbreviations: AFR, African; AMR, American; EAS, East Asian; EUR, European; SAS, South Asian.

In total, 18 articles were collected from the PubMed and WanFang databases via a literature search using different combinations of key words. As shown in Figure 1, 12 articles were excluded (two were duplications, 10 were irrespective articles). Finally, six different articles were included in current meta-analysis (Figure 1). In total, there were 2405 cases and 2607 controls. Study characteristics from the published studies on the relationship between the *CTLA-4* rs231775 G/A SNP and RPL risk are summarized in Table 1. In all the studies, the controls were women under normal pregnancy. Except one study, all studies were consistent with HWE. Finally, we checked the minor allele frequency (MAF) reported for the five main worldwide populations in the 1000 Genomes Browser [23]: East Asian (EAS), 0.3631; European (EUR), 0.3588; African (AFR), 0.3880; American (AMR), 0.4625; and South Asian (SAS), 0.3098 (Figure 2). The MAF in our analysis was 0.4033 and 0.5213 in the case and control group, respectively, both higher than the results in the EAS from1000 Genomes Browser database.

**Table 1:** Study characteristics from previous published studies on the association between *CTLA-4 gene* rs231775 polymorphism and RPL risk

Author	Year	Country	Ethnicity	SOC	Case	Control	Case			Control			HWE	Genotype	Case type	Control type	NOS
							AA	AG	GG	AA	AG	GG					
Wang [17]	2005	China	Asian	HB	168	117	20	66	82	17	61	39	0.38	PCR-RFLP	Unexplained RSA	Normal pregnancy	7
Chai [12]	2010	China	Asian	HB	233	224	28	101	104	35	95	94	0.18	PCR-RFLP	RSA	Normal pregnancy	7
Fan [13]	2018	China	Asian	HB	1284	1046	101	518	665	143	488	415	0.98	PCR-RFLP	RSA	Normal pregnancy	8
Gupta [14]	2012	India	Asian	HB	300	500	140	121	39	227	233	40	0.06	PCR-RFLP	RM	Normal pregnancy	7
Nasiri [16]	2016	Iran	Asian	HB	120	120	94	23	3	68	45	7	0.90	PCR-RFLP	RPL	Normal pregnancy	7
Misra [15]	2016	India	Asian	HB	300	600	105	135	60	264	288	48	0.01	PCR-RFLP	Idiopathic RM	Normal pregnancy	7

Abbreviations: HB, hospital based; HWE: Hardy–Weinberg equilibrium of control group; NOS: Newcastle–Ottawa scale; PCR-RFLP: polymerase chain reaction followed by restriction fragment length polymorphism; SOC; source of control.

### Quantitative synthesis

**Figure 3:**
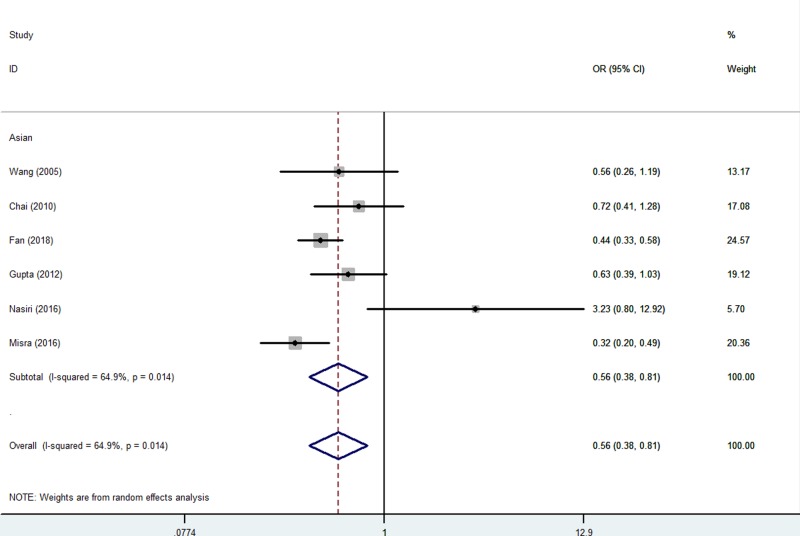
Forest plot of RPL risk associated with *CTLA-4* gene rs231775 polymorphism (AA vs. GG) in the whole The squares and horizontal lines correspond to the study-specific OR and 95% CI. The area of the squares reflects the weight (inverse of the variance). The diamond represents the summary OR and 95% CI.

There was significantly decreased association between the *CTLA-4* rs231775 G/A SNP and RPL risk susceptibility (AA vs. GG: OR = 0.56, 95% CI = 0.38–0.81, *P*_heterogeneity_=0.014, *P*=0.002, Figure 3, AG vs. GG: OR = 0.61, 95% CI = 0.46–0.80, *P*_heterogeneity_=0.034, *P*<0.001, and AA+AG vs. GG: OR = 0.61, 95% CI = 0.45–0.82, *P*_heterogeneity_=0.008, *P*=0.001) (Table 2).

**Table 2:** Total and stratified analysis of *CTLA-4 gene* rs231775 polymorphism and RPL risk

Total	OR(95% CI) *P*_h_ *P* genetic model
A-allele vs. G-allele	0.85(0.66–1.09)0.000 0.193 random model
AA vs. GG	0.56(0.38–0.81)0.014 0.002 random model
AG vs. GG	0.61(0.46–0.80)0.034 0.000 random model
AA+AG vs. GG	0.61(0.45–0.82)0.008 0.001 random model
AA VS. AG+GG	0.90(0.61–1.33)0.000 0.597 random model
Ethnicity subgroup	
China	OR(95% CI) *P*_h_ *P* genetic model
A-allele vs. G-allele	0.69(0.62–0.77)0.000 0.000 fixed model
AA vs. GG	0.49(0.39–0.63)0.294 0.002 fixed model
AG vs. GG	0.68(0.59–0.80)0.126 0.000 fixed model
AA+AG vs. GG	0.64(0.55–0.74)0.128 0.000 fixed model
AA VS. AG+GG	0.59(0.47–0.75)0.406 0.000 fixed model
Ethnicity subgroup	
Non-China	OR(95% CI) *P*_h_ *P* genetic model
A-allele vs. G-allele	1.06(0.61–1.84)0.000 0.834 random model
AA vs. GG	0.68(0.28–1.68)0.003 0.405 random model
AG vs. GG	0.46(0.34–0.63)0.237 0.000 fixed model
AA+AG vs. GG	0.60(0.29–1.23)0.015 0.166 random model
AA VS. AG+GG	1.20(0.64–2.26)0.000 0.573 random model

*P*_h_: value of *Q*-test for heterogeneity test; *P*: *Z*-test for the statistical significance of the OR

**Figure 4:**
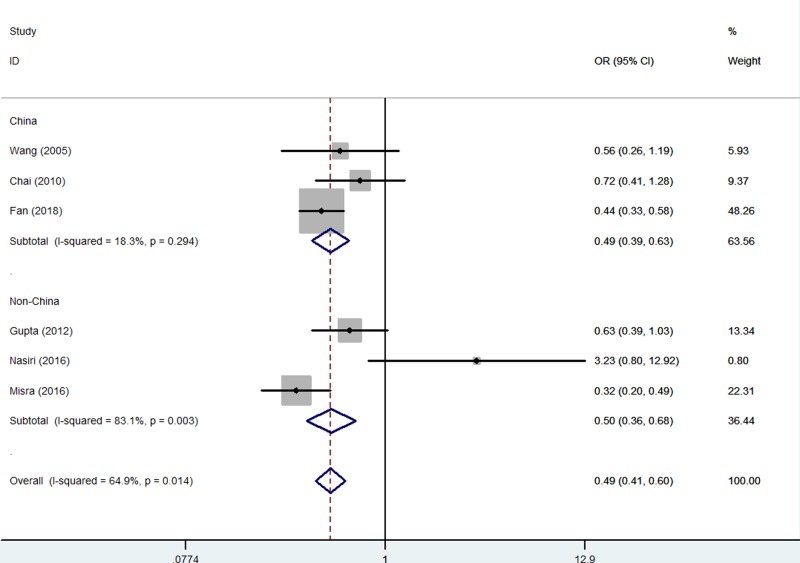
Forest plot of RPL risk associated with *CTLA-4* gene rs231775 polymorphism (AG vs. GG) in China population The squares and horizontal lines correspond to the study-specific OR and 95% CI. The area of the squares reflects the weight (inverse of the variance). The diamond represents the summary OR and 95% CI.

**Figure 5:**
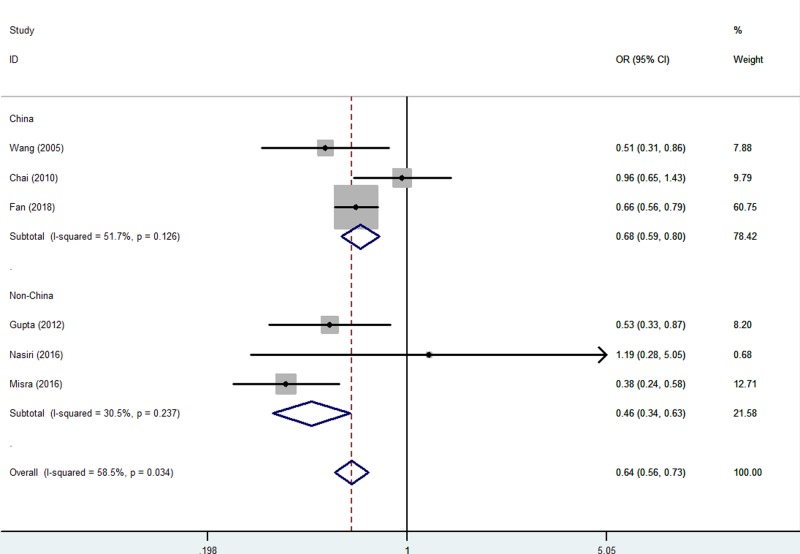
Forest plot of RPL risk associated with *CTLA-4* gene rs231775 polymorphism (AG vs. GG) in non-China population The squares and horizontal lines correspond to the study-specific OR and 95% CI. The area of the squares reflects the weight (inverse of the variance). The diamond represents the summary OR and 95% CI.

When studies were stratified according to ethnicity, there was also similar association found both in China (e.g. A-allele vs. G-allele: OR = 0.69, 95% CI = 0.62–0.77, *P*_heterogeneity_<0.001, *P*<0.001, AG vs. GG: OR = 0.68, 95% CI = 0.59–0.80, *P*_heterogeneity_=0.126, *P*<0.001, Figure 4, and AA vs. AG+GG: OR = 0.59, 95% CI = 0.47–0.75, *P*_heterogeneity_=0.406, *P*<0.001) and non-China risk (AG vs. GG: OR = 0.46, 95% CI = 0.34–0.63, *P*_heterogeneity_=0.237, *P*<0.001, Figure 5, Table 2).

### Sensitivity analysis and bias diagnosis

**Figure 6:**
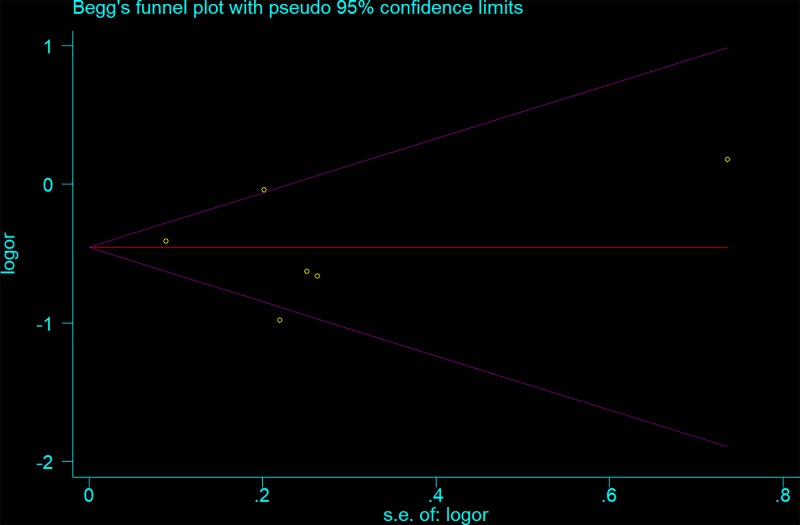
Sensitivity analysis between *CTLA-4* gene rs231775 polymorphism and tuberculosis risk (AG vs. GG)

**Figure 7:**
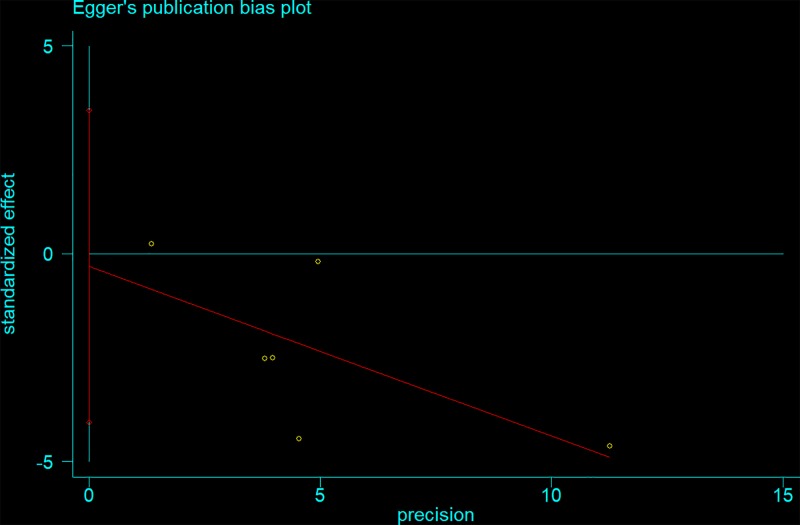
Begg’s funnel plot for publication bias test (AG vs. GG) Each point represents a separate study for the indicated association. Log [OR], natural logarithm of OR. Horizontal line, mean effect size.

**Figure 8:**
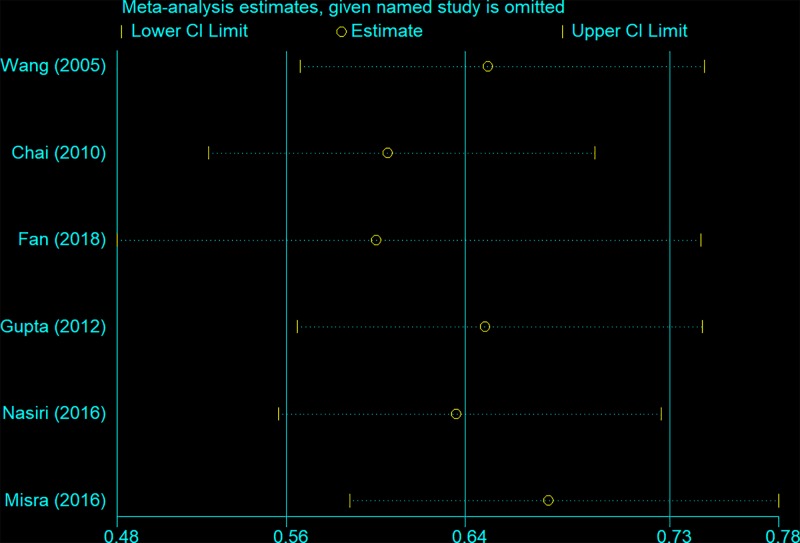
Egger’s publication bias plot (AG vs. GG)

We used a sensitivity analysis to determine whether modifying the meta-analysis inclusion criteria affected the results. No other single study influenced the summary OR qualitatively (Figure 6). Egger’s and Begg’s tests were performed to assess publication bias and the funnel plot symmetry was examined. Finally, no proof of publication bias was obtained (e.g. AG vs. GG: *t* = −0.23, *P*=0.892 for Egger’s test; and *z* = −0.19, *P*=0.851 for Begg’s test; Figures 7 and 8, Table 3).

**Table 3:** Publication bias tests (Begg’s funnel plot and Egger’s test for publication bias test) for *CTLA-4 gene* rs231775 polymorphism

Egger’s test						Begg’s test	
Genetic type	Coefficient	Standard error	*t*	*P-*value	95% CI of intercept	*z*	*P-*value
A-allele vs. G-allele	1.093	3.328	0.33	0.759	(−8.147,10.334)	0.75	0.452
AG vs. GG	−0.312	1.354	−0.23	0.892	(−4.072,3.446)	−0.19	0.851
AA vs. GG	0.422	1.461	0.29	0.787	(−3.634,4.479)	0.94	0.348
AA+AG vs. GG	−0.339	1.436	−0.24	0.825	(−4.327,3.647)	0	1
AA vs. AG+GG	2.753	2.308	1.19	0.299	(−3.655,9.162)	0.75	0.452

### Gene–gene interaction of online analysis

**Figure 9:**
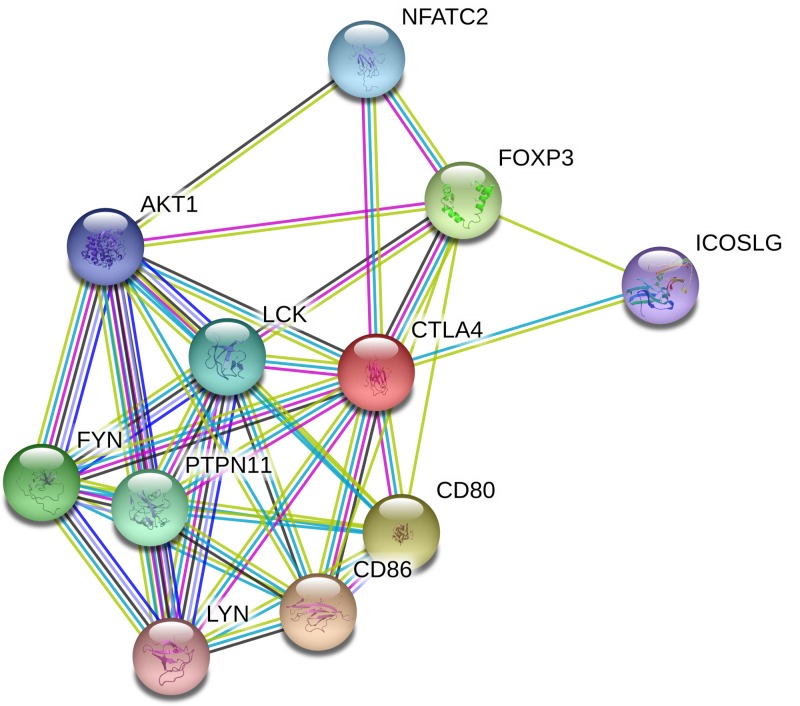
Human *CTLA-4* interactions network with other genes obtained from String server At least 10 genes have been indicated to correlate with *CTLA-4* gene. Abbreviations: AKT1, V-akt murine thymoma viral oncogene homolog 1; CD80, CD80 molecule; CD86, CD86 molecule; FOXP3, Forkhead box p3; FYN, FYN oncogene related to SRC, FGR, YES; ICOSLG, inducible T-cell co-stimulator ligand; LCK, lymphocyte-specific protein tyrpsine kinase; LYN, V-yes-1 Yamaguchi sarcoma viral-related oncogene homolog; NFATC2, nuclear factor of activated T-cells, cytoplasmic, calcineurin-dependent 2; PTPN11, protein tyrosine phosphatase, non-reveptor type 11.

String online server indicated that MTR gene interacts with numerous genes. The network of gene–gene interaction has been illustrated in Figure 9.

## Discussion

RPL is a common pregnancy complication affecting 1–3% of couples trying to conceive. Successful pregnancy is a result of maintaining the semi-allograft fetus from maternal immune responses [24]. The decision between tolerance and immunity determines the fate of each pregnancy. A network composed of different immune cells, numerous cytokines, growth factors, and adhesion molecules collaborate to reach the best outcome [25]. T_reg_ cells are cellular components of natural self-tolerance and seem to reduce the chance of pregnancy failure by providing the tolerant environment in the endometrium, where a successful implantation can occur [26]. Confirming evidence, in this regard, came from a significant reduction in circulating and deciduas T_reg_ cells among women with RPL [10,27]. One of the proposed mechanisms is the *CTLA-4*-dependent pathway using anti-*CTLA-4*-mAb disrupts the T_reg_ activity *in vivo*, in which T_reg_ provide this tolerance against the fetus [28].

To combine the importance of genetic etiology of RPL, it makes sense to deep study the *CTLA-4* gene polymorphisms. Rs231775 variant is one of common polymorphisms in *CTLA-4* gene. To our best of knowledge, it is the first time to select all published articles to analyze the association between *CTLA-4* gene rs231775 polymorphism and RPL susceptibility. In current study, the major discover is that rs231775 may decrease RPL risk, in other words, individuals carrying A-allele may have a decrease association for RPL, or A-allele is a protective factor for RPL risk, on the other hand, the G-allele is a potential risk factor. We boldly guess that the A-allele may increase the expression of CTLA-4 protein, because CTLA-4 is a protective factor in promoting fetus toleration and RPL [17].

In addition, we used the online analysis system String to predict potential and functional partners (Figure 9). Finally, ten genes were predicted. The highest score of association was CD86 and CD80 (score = 0.999); however, ICOSLG and LYN had the lowest scores (0.963 and 0.949, respectively). CD86 and CD80 are both the natural B7 family ligands of *CTLA-4*, the level of CD86(+) was significantly higher in the RPL group than the normal pregnancy group [29]. Several observations have indicated that CD28/*CTLA-4* and CD86/CD80 are involved in the maternal–fetal immune regulation, which might be potentially useful to immunotherapy for human RPL [30]. Most studies were focused on the association between FOXP3 gene and RPL, including SNPs and immune regulation (such as T_reg_, CD4+CD25+, Th17) [13]. Karim et al. reported that several novel CNVs/genes (such as 14q32.33/AKT1) in chromosomal abnormalities were associated with RPL risk [31]. Above information predicted that CD86, CD80, FOXP3, and AKT1 may influence *CTLA-4* and regulate the RPL development, which may become intervention and treatment target genes in the future.

Limitations in the present meta-analysis include the suboptimal number of published studies for a comprehensive analysis. Second, interactions between different polymorphic loci of the same *CTLA-4* may modulate RPL risk, which should be included in future research and analysis. In addition, our meta-analysis was based on unadjusted estimates. A more precise analysis should be conducted if individual data are available to adjust for other covariates including age, sex, family history, environmental factors, endocrine abnormalities, each type of PL, and lifestyle.

In summary, in the present meta-analysis, a significant decreased association was found between the *CTLA-4* gene rs231775 SNP and RPL risk. To further confirm the results, larger scale case–control studies with different ethnic groups and multiple PL types are needed.

## Abbreviations

CI: confidence interval
*CTLA4*: cytotoxic T-lymphocyte antigen 4
HWE: Hardy–Weinberg equilibrium
ID: indoleamine 2,3-dioxygenase
MAF: minor allele frequency
NOS: Newcastle–Ottawa Scale
PL: pregnancy loss
RM: recurrent miscarriage
RSA: recurrent spontaneous abortion
SNP: single nucleotide polymorphism

## References

[1] Goddijn M., Elson J., Peramo B., Bender Atik R., Christiansen O., Kolte A. (2017) Guideline on the management of recurrent pregnancy loss. Eur. Soc. Human Reprod. Embryol. 2, 1–10 10.1007/978-3-319-27452-2

[2] Royal College Obstetricians and Gynaecologists (RCOG) (2011) The investigation and treatment of couples with recurrent first-trimester and second-trimester miscarriage. R. Coll. Obstet. Gynaecol. 17, 1–18

[3] Salat-Baroux J. (1988) [Recurrent spontaneous abortions]. Reprod. Nutr. Dev. 28, 1555–1568 10.1051/rnd:19881002 3073445

[4] (2013) Definitions of infertility and recurrent pregnancy loss: a committee opinion. Fertil. Steril. 99, 63, Practice Committee of American Society for Reproductive Medicine 10.1016/j.fertnstert.2012.09.023 23095139

[5] Jauniaux E., Farquharson R.G., Christiansen O.B. and Exalto N. (2006) Evidence-based guidelines for the investigation and medical treatment of recurrent miscarriage. Hum. Reprod. 21, 2216–2222 10.1093/humrep/del150 16707507

[6] Toth B., Wurfel W., Bohlmann M.K., Gillessen-Kaesbach G., Nawroth F., Rogenhofer N. (2015) Recurrent miscarriage: diagnostic and therapeutic procedures. Guideline of the DGGG (S1-Level, AWMF Registry No. 015/050, December 2013). Geburtshilfe Frauenheilkd 75, 1117–1129 10.1055/s-0035-1558299 26997666 PMC4795844

[7] Garrido-Gimenez C. and Alijotas-Reig J. (2015) Recurrent miscarriage: causes, evaluation and management. Postgrad. Med. J. 91, 151–162 10.1136/postgradmedj-2014-132672 25681385

[8] Kucharska A.M., Gorska E., Wasik M., Pyrzak B. and Demkow U. (2009) Expression of CD152 (CTLA-4) in children with autoimmune thyroiditis and +49 A/G polymorphism of exon 1 of the CTLA-4 gene. J. Physiol. Pharmacol. 60 Suppl 5, 77–80 20134044

[9] Sasaki Y., Sakai M., Miyazaki S., Higuma S., Shiozaki A. and Saito S. (2004) Decidual and peripheral blood CD4+CD25+ regulatory T cells in early pregnancy subjects and spontaneous abortion cases. Mol. Hum. Reprod. 10, 347–353 10.1093/molehr/gah044 14997000

[10] Jin L.P., Chen Q.Y., Zhang T., Guo P.F. and Li D.J. (2009) The CD4+CD25 bright regulatory T cells and CTLA-4 expression in peripheral and decidual lymphocytes are down-regulated in human miscarriage. Clin. Immunol. 133, 402–410 10.1016/j.clim.2009.08.009 19766059

[11] Shastry B.S. (2009) SNPs: impact on gene function and phenotype. Methods Mol. Biol. 578, 3–22 10.1007/978-1-60327-411-1_1 19768584

[12] Chai L. (2010) Association of the CTLA-4 Gene Polymorphism with the Recurrent Spontaneous Abortion. Master’s Degree Thesis, Nixia Medical University

[13] Fan Q., Zhang J., Cui Y., Wang C., Xie Y., Wang Q. (2018) The synergic effects of CTLA-4/Foxp3-related genotypes and chromosomal aberrations on the risk of recurrent spontaneous abortion among a Chinese Han population. J. Hum. Genet. 63, 579–587 10.1038/s10038-018-0414-2 29476189 PMC5915418

[14] Gupta R., Prakash S., Parveen F. and Agrawal S. (2012) Association of CTLA-4 and TNF-alpha polymorphism with recurrent miscarriage among North Indian women. Cytokine 60, 456–462 10.1016/j.cyto.2012.05.018 22727980

[15] Misra M.K., Mishra A., Phadke S.R. and Agrawal S. (2016) Association of functional genetic variants of CTLA4 with reduced serum CTLA4 protein levels and increased risk of idiopathic recurrent miscarriages. Fertil. Steril. 106, 1115–1123.e1116 10.1016/j.fertnstert.2016.06.011 27351445

[16] Nasiri M. and Rasti Z. (2016) CTLA-4 and IL-6 gene polymorphisms: risk factors for recurrent pregnancy loss. Hum. Immunol. 77, 1271–1274 10.1016/j.humimm.2016.07.236 27480842

[17] Wang X., Lin Q., Ma Z., Hong Y., Zhao A., Di W. (2005) Association of the A/G polymorphism at position 49 in exon 1 of CTLA-4 with the susceptibility to unexplained recurrent spontaneous abortion in the Chinese population. Am. J. Reprod. Immunol. 53, 100–105 10.1111/j.1600-0897.2004.00251.x 15790344

[18] Wells G., Shea B., O’Connell D., Robertson J., Peterson J., Welch V. (2011) The Newcastle–Ottawa Scale (NOS) for Assessing the Quality of Nonrandomised Studies in Meta-analyses. Ottawa Health Research Institute

[19] Higgins J.P. and Thompson S.G. (2002) Quantifying heterogeneity in a meta-analysis. Stat. Med. 21, 1539–1558 10.1002/sim.1186 12111919

[20] DerSimonian R. and Laird N. (1986) Meta-analysis in clinical trials. Control. Clin. Trials 7, 177–188 10.1016/0197-2456(86)90046-2 3802833

[21] Mantel N. and Haenszel W. (1959) Statistical aspects of the analysis of data from retrospective studies of disease. J. Natl. Cancer Inst. 22, 719–748 13655060

[22] Egger M., Davey Smith G., Schneider M. and Minder C. (1997) Bias in meta-analysis detected by a simple, graphical test. BMJ 315, 629–634 10.1136/bmj.315.7109.629 9310563 PMC2127453

[23] Shao H.B., Ren K., Gao S.L., Zou J.G., Mi Y.Y., Zhang L.F. (2018) Human methionine synthase A2756G polymorphism increases susceptibility to prostate cancer. Aging 10, 1776–1788 10.18632/aging.101509 30064122 PMC6075445

[24] Guerin L.R., Prins J.R. and Robertson S.A. (2009) Regulatory T-cells and immune tolerance in pregnancy: a new target for infertility treatment. Hum. Reprod. Update 15, 517–535 10.1093/humupd/dmp004 19279047 PMC2725755

[25] van Mourik M.S., Macklon N.S. and Heijnen C.J. (2009) Embryonic implantation: cytokines, adhesion molecules, and immune cells in establishing an implantation environment. J. Leukoc. Biol. 85, 4–19 10.1189/jlb.0708395 18784344

[26] Zenclussen A.C. (2006) Regulatory T cells in pregnancy. Springer Semin. Immunopathol. 28, 31–39 10.1007/s00281-006-0023-6 16838178

[27] Yang H., Qiu L., Chen G., Ye Z., Lu C. and Lin Q. (2008) Proportional change of CD4+CD25+ regulatory T cells in decidua and peripheral blood in unexplained recurrent spontaneous abortion patients. Fertil. Steril. 89, 656–661 10.1016/j.fertnstert.2007.03.037 17543960

[28] Read S., Greenwald R., Izcue A., Robinson N., Mandelbrot D., Francisco L. (2006) Blockade of CTLA-4 on CD4+CD25+ regulatory T cells abrogates their function in vivo. J. Immunol. 177, 4376–4383 10.4049/jimmunol.177.7.4376 16982872 PMC6108417

[29] Toldi G., Vasarhelyi B., Biro E., Fugedi G., Rigo J. Jr. and Molvarec A. (2013) B7 costimulation and intracellular indoleamine-2,3-dioxygenase expression in peripheral blood of healthy pregnant and pre-eclamptic women. Am. J. Reprod. Immunol. 69, 264–271 10.1111/aji.12069 23289444

[30] Wang X., Ma Z., Hong Y., Lu P. and Lin Q. (2006) Expression of CD28 and cytotoxic T lymphocyte antigen 4 at the maternal-fetal interface in women with unexplained pregnancy loss. Internat. J. Gynaecol. Obstet. 93, 123–129 10.1016/j.ijgo.2006.01.027 16564528

[31] Karim S., Jamal H.S., Rouzi A., Ardawi M.S.M., Schulten H.J., Mirza Z. (2017) Genomic answers for recurrent spontaneous abortion in Saudi Arabia: an array comparative genomic hybridization approach. Reprod. Biol. 17, 133–143 10.1016/j.repbio.2017.03.003 28431992

